# QoS-Driven Adaptive Trust Service Coordination in the Industrial Internet of Things

**DOI:** 10.3390/s18082449

**Published:** 2018-07-27

**Authors:** Jin Qi, Zian Wang, Bin Xu, Mengfei Wu, Zian Gao, Yanfei Sun

**Affiliations:** 1 College of Internet of Things, Nanjing University of Posts and Telecommunications, Nanjing 210003, China; b15070611@njupt.edu.cn (Z.W.); xubin2013@njupt.edu.cn (B.X.); 1017010305@njupt.edu.cn (M.W.); 2 Jiangsu Engineer Research Center of Communication and Network Technology, Nanjing University of Posts and Telecommunications, Nanjing 210003, China; 3 College of Telecommunications and Information Engineering, Nanjing University of Posts and Telecommunications, Nanjing 210003, China; ziangaoann@yahoo.com

**Keywords:** industrial internet of things, trust service, adaptive coordination, QoS-driven, multi-objective gray-wolf optimization, blockchain

## Abstract

The adaptive coordination of trust services can provide highly dependable and personalized solutions for industrial requirements in the service-oriented industrial internet of things (IIoT) architecture to achieve efficient utilization of service resources. Although great progress has been made, trust service coordination still faces challenging problems such as trustless industry service, poor coordination, and quality of service (QoS) personalized demand. In this paper, we propose a QoS-driven and adaptive trust service coordination method to implement Pareto-efficient allocation of limited industrial service resources in the background of the IIoT. First, we established a Pareto-effective and adaptive industrial IoT trust service coordination model and introduced a blockchain-based adaptive trust evaluation mechanism to achieve trust evaluation of industrial services. Then, taking advantage of a large and complex search space for solution efficiency, we introduced and compared multi-objective gray-wolf algorithms with the particle swarm optimization (PSO) and dragonfly algorithms. The experimental results showed that by judging and blacklisting malicious raters quickly and accurately, our model can efficiently realize self-adaptive, personalized, and intelligent trust service coordination under the given constraints, improving not only the response time, but also the success rate in coordination.

## 1. Introduction

With the rapid development of the internet of things, a series of national strategies such as German Industry 4.0 [1], the Advanced Manufacturing Partner Program of the United States [2], and China Manufacturing 2025 [3] have been proposed in succession. Under this background, the industrial internet of things (IIoT) came into being, becoming an important promoter of the intelligent transformation of the global industrial system. The IIoT realizes a flexible configuration of raw material production, on-demand execution of manufacturing processes, reasonable optimization of manufacturing technique, and rapid adaptation of the manufacturing environment through network interconnection, data interoperability, and system interoperability of industrial resources to realize efficient use of resources, thus building a new service-driven industrial system [4]. The core capability of the IIoT is its service capability. Everything as a service (XaaS) has become the ultimate goal of the IIoT. The new service-oriented model of intelligent industry is the research hotspot on which academia and industry presently focus.

The studies of the IIoT have been conducted by domestic and foreign scholars and research teams, and some achievements have been made. The main achievements focus on the architecture of the IIoT [5,6,7], communication protocol [8], topology optimization [9], data mining [10], and system implementation [11]. Recently, great efforts have been made to improve the greenness of various architectures in the IIoT. Wang et al. proposed an energy-efficient architecture in the green IIoT [12]. He et al. proposed a green resource allocation method based on deep reinforcement learning in the content-centric IoT [13]. However, the implementation of IoT trust, personality, and intelligent services in a complex environment is the driving purpose of the IIoT. Therefore, research on the IIoT should pay more attention to the adaptive trust service coordination driven by quality of service (QoS) requirements. The original model of service coordination aims primarily at selecting an appropriate set of concrete services and composing them to achieve the QoS and quality of experience (QoE) goals. For example, He et al. proposed a QoE-driven big data structure for smart city [14]. Ma et al. proposed a coordination scheme using the knowledge for deriving optimal QoS-driven coordination solutions [15]. Wang et al. proposed a trustworthy crowdsourcing model in the social internet of things (SIoT) [16]. Chen and Paik proposed a quality-driven coordination method based on a social service network [17]. Qi et al. proposed a knowledge based differential evolution algorithm to solve cloud computing Web service coordination [18]. However, none of the above methods have been combined with the IIoT environment and considered the trust of the service. Xue et al. proposed a task-driven manufacturing cloud service (MCS) proactive discovery and optimal configuration method to realize full-scale sharing, on-demand use, and collaborative configuration of manufacturing resources [19]. Zhang et al. proposed a QoS-driven manufacturing service adaptation method based on a networked collaboration model, which can effectively implement specific cross-enterprises service adaptation [20]. Both methods consider the IIoT environment, but not the trust of service. However, as the number of IIoT access services increases, plenty of malicious service providers also get involved in the network, which can launch attacks to benign service providers and threaten the balance and rationality of the IIoT. Thus, trust evaluation of malicious industrial services becomes extremely necessary. Noor proposed a reputation-based trust management method for cloud services that could effectively protect cloud services against malicious users [21]. However, its reputation management method lacks self-adaptability. Chen proposed an adaptable and extendable trust management model to support social IoT service adaptation [22]. However, the author paid more attention to the trust management model and did not consider effective QoS-driven Pareto service allocation. With the increasing number of industrial access devices, the traditionally subjective way of trust evaluation cannot meet the demand for objective evaluation of massive services. In recent years, an increasing number of scholars have focused on using blockchain to optimize trust evaluations. Fu and Fang proposed the concept of computing the trust value of nodes under the background of trusted computing, but they did not consider the efficiency of the allocation of computing tasks [23]. Huang et al. proposed an IoT data trusted exchange based on blockchain, but they did not evaluate the trust value of the nodes in the IoT, which is unadaptable [24]. The purpose of QoS-driven adaptive trust service coordination is to meet the QoS-driven service requirement of the IIoT. Under certain conditional constraints, all types of industrial services will generate corresponding service adaptation implementation paths in accordance with certain rules to achieve the optimal allocation and efficient use of industrial resources and realize the Pareto-efficient allocation of trust industrial resources. Therefore, investigations on the adaptive trust service coordination driven by QoS requirements are necessary.

This paper aims to establish a QoS-driven self-adaptation trust service coordination method in the IIoT environment. First, we establish a Pareto-efficient IIoT trust service coordination model and a self-adaptive trust evaluation model and introduce the multi-objective gray-wolf optimizer (MOGWO), which uses its flexible and balanced mechanism to adapt to the global and local exploration and development capabilities. The MOGWO can effectively deal with a large and complex search space. The experimental results show that the model is effective and accurate and can effectively achieve the self-adaptation of the given constraints under the conditions of personalized, intelligent, and credible service coordination.

The contributions of this article are three-fold:

1. A QoS-driven IIoT adaptive trust service coordination method is proposed that realizes the multi-index evaluation adaptation to the response time, availability, throughput, and reliability by collecting data corresponding to various QoS indicators in the IIoT through industrial sensor networks. The coordination process satisfies the Pareto-effective allocation idea, which can effectively realize the optimal allocation of the IIoT trust service resources.
2. A blockchain-based adaptive trust evaluation model for the IIoT is proposed. Through the P2P ratings on coordination degree among service partners and adaptive filtering algorithms, the problem of possible attacks launched by malicious service providers in the IIoT is solved, which can improve the effectiveness, stability, and accuracy of assessments.
3. An improved MOGWO is proposed to solve the QoS-driven self-adaptation trust service coordination fit method. Based on the basic wolf algorithm, a Pareto archive and update mechanism of storage space similarity in the Pareto solution set are introduced. This method enables adaptive use cases to be learned and developed, which can effectively improve the diversity of the final approximate Pareto-optimal frontier—the set of all Pareto-effective [25] solutions.

The remainder of this paper is organized as follows: The second section provides a description of the Pareto-efficient allocation model for the IIoT trust service coordination. The third section introduces the MOGWO for self-adaptive service coordination in the IIoT. The fourth section provides experimental verification of the effectiveness of the adaptive coordination model and the trust evaluation model and makes a comparative analysis. The fifth section presents the conclusion.

## 2. Problem Description

**Figure 1:**
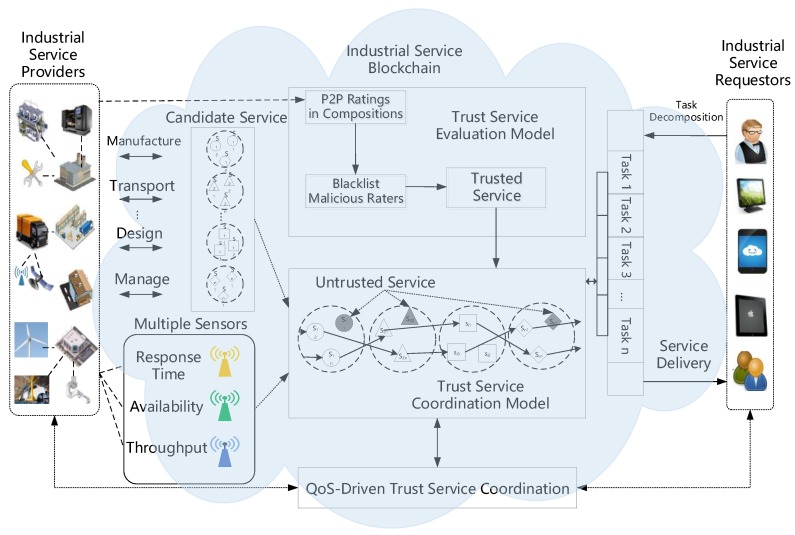
Adaptive trust service coordination framework in the industrial internet of things (IIoT). QoS—quality of service.

At present, effectively realizing a personalized and intelligent trust service coordination of autonomous adaptation is difficult for traditional industrial processes. Therefore, this paper proposes a QoS-driven adaptive trust service coordination framework in the IIoT. The paper integrates adaptive trust evaluation and intelligent service optimization into a QoS guarantee of the IIoT and organically integrates the IoT with the traditional industrial processes, which is beneficial in terms of reducing industrial costs and resource consumption, meeting the needs of personalized service users, and improving the quality of industrial service coordination under the given constraints. The framework of the adaptive trust service coordination is shown in Figure 1.

### 2.1. QoS-Driven Adaptive Trust Service Coordination Model in the IIoT

QoS-driven service coordination in the IIoT is the process of optimally allocating IIoT resources and is a way to evaluate the QoS based on the non-functional characteristics of industrial IoT services, including response time, availability, throughput, trust, latency, and other factors. Because the traditional linear weighted service adaptation model cannot accurately measure the above indicators, this paper selects Pareto-effective thought, which meets the coordination process requirements for evaluating multiple indicators and chooses the response time, availability, throughput, and trust as the evaluation index:

(1) $$QoS=\left\{Response\;Time,Availability,Throughput,Trust\right\}=\left\{ReT_{i},Ava_{i},Thr_{i},T_{i}\right\}$$

where $ReT_{i}$ is the service response time, $Ava_{i}$ is the availability, $Thr_{i}$ is the throughput, and $T_{i}$ is the trust. When the total service GS can be divided into $GS=\left\{SS_{1},SS_{2},\ldots ,SS_{n}\right\}$, the overall response time is $\overset{n}{\underset{i=1}{\sum }}ReT_{i}$. The total availability is $\overset{n}{\underset{i=1}{\prod }}Ava_{i}$. The throughput is $\min\left\{Thr_{1},Thr_{2},\ldots ,Thr_{n}\right\}$, and the total trust is $\overset{n}{\underset{i=1}{\sum }}T_{i}$. The service coordination model for the IIoT based on the adaptive trust evaluation established in this paper is as follows:

(2) $$QoS=\left(\overset{n}{\underset{i=1}{\sum }}ReT_{i},\overset{n}{\underset{i=1}{\prod }}Ava_{i},\min\left\{Thr_{1},Thr_{2},\ldots ,Thr_{n}\right\},\overset{n}{\underset{i=1}{\sum }}T_{i}\right)$$

The maximum response time accepted by a GS is RT, and the minimum acceptable throughput is TH. The acceptable availability is A, the minimum allowed trustworthiness is T, and the constraint is:

(3) $$\{\begin{array}{c} RT\leq \overset{n}{\underset{i=1}{\sum }}ReT_{i}\\ TH\leq \min\left\{Thr_{1},Thr_{2},\ldots ,Thr_{n}\right\}\\ A\leq \overset{n}{\underset{i=1}{\prod }}Ava_{i}\\ \;T\leq \overset{n}{\underset{i=1}{\sum }}T_{i} \end{array}$$

### 2.2. Adaptive Trust Evaluation Model Based on Blockchain in the IIoT

The dynamic variety of the IIoT brings great challenges to the trust of the service. Being static and single, the traditionally subjective assessment methods have difficulty meeting the trust service coordination of industrial processes. Therefore, this paper proposes a blockchain-based self-adaptation trust evaluation model based on the coordination degree. 

**Figure 2:**
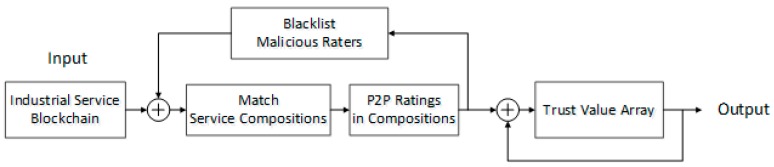
Adaptive trust evaluation model based on the IIoT service compositions.

As shown in Figure 2, the service providers in the IIoT service compositions perform peer-to-peer (P2P) ratings with each other based on the coordination degree. Through the trust evaluation mechanism, a trust value array is obtained. At the same time, the malicious raters in the service composition are filtered according to an adaptive filtering mechanism, thus blocking malicious ratings. The inability to tamper with the blockchain can guarantee the correctness of the trust value evaluated under this mechanism. Second, the great confidentiality and anonymity of the blockchain allow the raters to observe the ratings of other raters in the service composition during the rating process, thus ensuring the authenticity and objectivity of the ratings.

**Figure 3:**
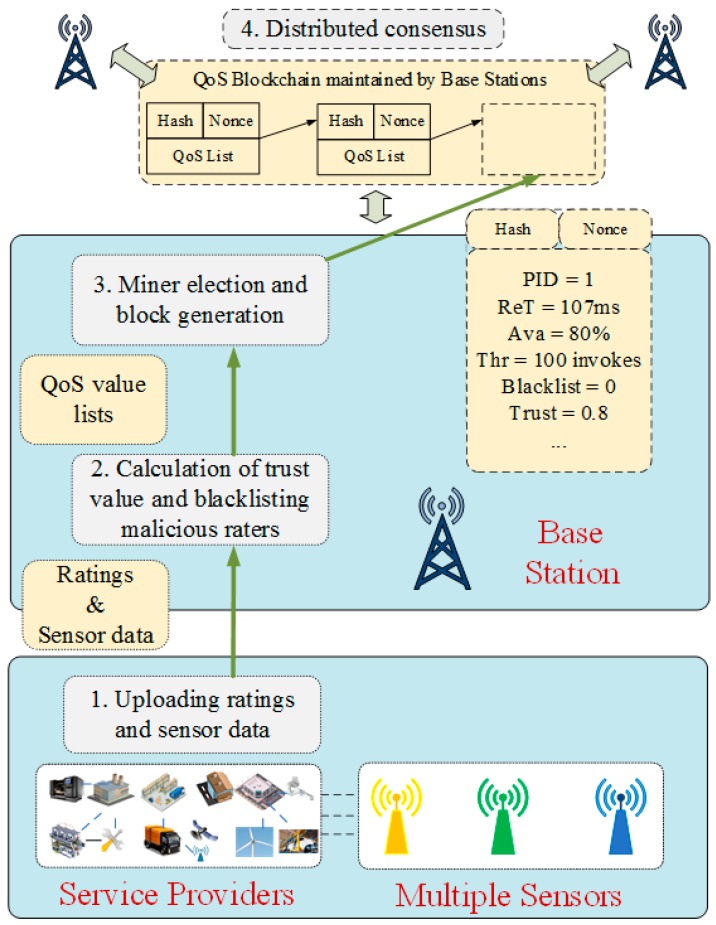
System design of blockchain-based adaptive trust evaluation. PID—identification number of service providers; ReT—service response time; Ava—availability; Thr—throughput.

As illustrated in Figure 3, the main procedures of blockchain-based adaptive trust evaluation can be divided into three steps. (1) Uploading ratings and sensor data; (2) calculation of trust value and blacklisting malicious raters; (3) miner election and block generation; and (4) distributed consensus.

#### 2.2.1. Attack Model

● Self-promoting attacks

Malicious service providers increase their trust value by promoting themselves (by providing good ratings for themselves) to increase their possibility of being selected as service providers in the IIoT. After being selected as a service provider, malicious service providers will provide inferior services.

● Bad-mouthing and ballot-stuffing attacks

Malicious service providers can cooperate with other malicious service providers to provide good ratings for each other. At the same time, they provide bad ratings for benign service providers, greatly enhancing their own trust value and affecting the trust value of benign service providers. Thereby, malicious service providers can greatly increase their own possibility of being selected as a service provider. At the same time, this method can effectively avoid the monitoring of most traditional credit systems.

● Opportunistic service attacks

The malicious service provider increases its trust value to a higher level by providing a certain amount of high-quality and dependable services at first. At the same time, the provider sometimes provides inferior services to obtain additional profits on the basis of maintaining its own trust value at a relatively high level.

● Hybrid attacks

The malicious service providers perform the abovementioned bad-mouthing and ballot-stuffing attacks and opportunistic service attacks simultaneously. This hybrid attack makes the trust evaluation structure in the IIoT more complex and requires a higher demand for filtering algorithms.

#### 2.2.2. Trust Evaluation Mechanism

In a service composition, there is always a comparatively large amount of service providers. Therefore, the coordination degree of the service providers in the service composition is a key factor in the trust evaluation process. We start with the coordination degree of the service providers in the service composition to evaluate the trust value of the service providers. In the service composition, the comprehensive rating $R_{i}$ obtained by the service provider $P_{i}$ is calculated using the ratings of the other service providers. In Equation (4), we propose a comprehensive rating mechanism in the service composition that contains n service providers:

(4) $$R_{i}=\frac{\sum_{j=1}^{n-1}T_{j}*R_{j,i}}{\sum_{j=1}^{n-1}T_{j}}$$

where $T_{j}$ is the personal trust of the rater, which is a real number in the range of [−1,1] where −1, 0, and 1 indicate distrust, ignorance, and complete trust, respectively. $R_{j,i}$ is the rating of $P_{j}$ to $P_{i}$, which is also a real number in the range of [−1,1]. In this mechanism, the service provider $P_{j}$ cannot rate itself. Therefore, this mechanism can avoid self-promoting attacks.

Next, in Equation (5), we propose a mechanism for updating trust based on comprehensive ratings in service composition.

(5) $$T_{i}\left(t\right)=\{\begin{array}{c} \min[1,T_{i}\left(t-\Delta t\right)+\alpha F\left(\Delta R_{i}\right)],\;R_{i}\geq T_{i}\left(t-\Delta t\right)\\ max\left[-1,T_{i}\left(t-\Delta t\right)-\beta F\left(\Delta R_{i}\right)\right],\;R_{i}<T_{i}\left(t-\Delta t\right) \end{array}$$

where $T_{i}\left(t\right)$ is the evaluated trust value of the service provider $P_{i}$, $T_{i}\left(t-\Delta t\right)$ is the trust value of the service provider $P_{i}$ since the last update, and *α* and *β* are the parameters in the trust evaluation. We hope to achieve a slower increase and a faster decrease in trust by setting *α* and *β*. *F*(*x*) is a trust adjustment function when the trust value increases and decreases. We define *F*(*x*) and $\Delta R_{i}$ in Equations (6) and (7), respectively.

(6) $$F\left(x\right)=\cos\left(\pi +\frac{\pi }{4}x\right)+1$$

(7) $$\Delta R_{i}=\left|R_{i}-T_{i}\left(t-\Delta t\right)\right|$$

where $\Delta R_{i}$ is in the range of [0,2] and, consequently, $F\left(\Delta R_{i}\right)$ is in the range of [0, 1]. We use the cos(x) function to design F(x) in order to achieve the effect that, when the deviation between the comprehensive $R_{i}$ obtained by $P_{i}$ and its trust value $T_{i}\left(t-\Delta t\right)$ is not large, the change in the trust value is relatively small, whereas the change in trust value is more obvious when the deviation is large.

#### 2.2.3. Malicious Rater Filtering Mechanism

Through the anonymity and tamper-resistance of blockchain, our mechanism filter and blacklist malicious raters are based on anonymous P2P ratings in service compositions. According to the characteristic that the number of malicious service providers in the IIoT is fewer than that of the SIoT (social internet of things), we proposed a malicious rater filtering mechanism for the IIoT. To be specific, the mechanism is described by Algorithm 1 as follows.
**Algorithm 1.** Adaptive malicious rating filter algorithm based on rating sets**Input:** We use the random function to select 30% nodes, which act as malicious raters and initialize 10 service compositions that contain 10 to 20 service providers in each interaction round. Malicious raters launch bad mouthing attacks and ballot stuffing attacks while the ratings given by benign raters are random real numbers in the range of [0.7,0.9]. Thus, the rating set $\left\{R_{1,\;i},\;R_{2,\;i},\;\ldots ,\;R_{n-1,\;i}\right\}$ obtained by service provider $P_{i}$ from its partners can be derived. **Output:** A blacklist of malicious raters is judged in the final.1 for each service composition 2  for each $P_{i}$
3   $C_{i}=C_{i}+1$
4  end for 5  for each rating set $\left\{R_{1,i},\;R_{2,i},\;\ldots ,\;R_{n-1,i}\right\}$ in the composition6   Calculate the average rating $\bar{R_{i}}$ and the variance $\sigma_{i}$ of the set 7   Calculate the deviation set $\left\{E_{1,i},\;E_{2,i},\ldots ,E_{n-1,i}\right\}$
8   if $\sigma_{i}<0.1$
9    Select the rater $P_{j}$ which has the largest deviation 10    $M_{j}=M_{j}+1$
11   else 12    for each $E_{j,\;i}$
13     if $E_{j,\;i}>H\left(\sigma_{i}\right)$
14      $M_{j}=M_{j}+1$
15     end if 16    end for 17   end if 18   Update $\left\{MP_{1},MP_{2},\ldots ,MP_{n}\right\}$
19   for each $MP_{i}$
20    if $MP_{i}>\;\hat{\;MP}$
**&**
$C_{i}>\;\hat{C}$
21     add $P_{i}$ to the blacklist 22    end if 23   end for 24  end for 25 end for
where H(x) is an adaptive function and $\hat{\;MP}$ and $\hat{C}$ are the thresholds of $MP_{i}$ and $C_{i}$, respectively. We will study the performance of different $H\left(x\right),\hat{\;MP}\;\&\;\hat{C}$ in the experimental section to achieve the effect of filtering malicious raters effectively and accurately.

## 3. Trust Service Coordination Using Multi-Objective Gray-Wolf Optimization in the IIoT

### 3.1. Gray-Wolf Optimizer

The gray-wolf optimizer algorithm (GWO) [26,27] is a novel swarm intelligence algorithm that simulates the division of labor and foraging behavior in the wolf cluster. The goal of this algorithm is to realize that various wolves (Canis lupus) cooperate with each other to better maximize the success rate of capturing prey, finding the optimal solution to the optimization problem. In this intelligence behavior, there are four types of wolves in the gray-wolf cluster, namely, alpha, beta, delta, and omega. Among them, cluster leadership, surrounding prey, and hunting behavior are the three main behaviors of gray wolves catching prey:

(1) Social hierarchy. Each wolf pack has a strict hierarchy. The individual with the best fitness value becomes wolf α, whereas individuals with the second and third largest fitness values become wolf β and wolf δ, respectively. The remaining individuals are named wolves ω. In the MOGWO algorithm, hunting is led by wolves α, β, and δ and wolf ω is responsible for following the three wolves to find the optimal solution.

(2) Encircling prey. Gray wolves first verify the distance between the prey and themselves:

(8) $$\vec{D}=\left|\vec{C}\cdot \vec{x_{p}}\left(t\right)-\vec{X}\left(t\right)\right|$$

(9) $$\vec{C}=\;2\vec{r_{2}}$$

where *t* is the current iteration, $\vec{C}$ is the vector of coefficients, $\vec{x_{p}}\left(t\right)$ is the vector of the prey’s position, $\vec{X}$ is the vector of the gray wolves, and $\vec{r_{2}}$ is a random vector in the range of [0,1].

Then, the wolves update their positions based on their distance from their prey:

(10) $$\vec{X}\left(t+1\right)=\vec{x_{p}}\left(t\right)-\vec{A}\cdot \vec{D}$$

(11) $$\vec{A}=2\vec{a}\cdot \vec{r_{1}}-\vec{a}$$

where $\vec{a}$ decreases linearly from 2 to 0 during the iteration process, and $\vec{r_{1}}$ is a random vector in the range of [0, 1].

(3) Hunting. In the gray-wolf algorithm, the location of the prey is constantly changing. The wolves do not know the specific location of the prey. To capture the prey, three wolves with better fitness values are selected to locate the prey. The mathematical model can be described as follows:

(12) $$\vec{D_{\propto }}=\left|\vec{C_{1}}\cdot \vec{x_{\propto }}\left(t\right)-\vec{X}\right|,\;\vec{x_{1}}=\vec{x_{\alpha }}\left(t\right)-\vec{A_{1}}\cdot \vec{D_{\alpha }}$$

(13) $$\vec{D_{\beta }}=\left|\vec{C_{2}}\cdot \vec{x_{\beta }}\left(t\right)-\vec{X}\right|,\;\vec{x_{2}}=\vec{x_{\beta }}\left(t\right)-\vec{A_{2}}\cdot \vec{D_{\beta }}$$

(14) $$\vec{D_{\delta }}=\left|\vec{C_{3}}\cdot \vec{x_{\delta }}\left(t\right)-\vec{X}\right|,\;\vec{x_{3}}=\vec{x_{\delta }}\left(t\right)-\vec{A_{3}}\cdot \vec{D_{\delta }}$$

(15) $$\vec{X}\left(t+1\right)=\left(\vec{x_{1}}+\vec{x_{2}}+\vec{x_{3}}\right)/3$$

(4) The attack process (exploitation) is as follows:

When the prey stops moving, the wolf hunts them by attacking. To simulate approaching the prey mathematically, we reduce the value of $\vec{a}$. Note that the fluctuation range of $\vec{A}$ is also reduced. In other words, $\vec{A}$ is the random value in the interval [−2a,2a] where a decreases from 2 to 0 during the iteration. When the random value of $\vec{A}$ is [−1,1], the search agent’s next position can be any position between its present position and the prey’s position.

(5) The search process (exploration) is as follows:

Gray wolves search mostly according to the position of alpha, beta, and delta. They diverge from each other to search for prey and converge to attack prey. To simulate divergence mathematically, we use $\vec{A}$, a random value greater than 1 or less than −1, in order to force the search agent to disagree with the prey, thereby emphasizing the exploration and allowing the MOGWO algorithm to search globally.

### 3.2. Adaptive Trust Service Coordination of IIoT Based on the Multi-Objective Gray-Wolf Algorithm

To make the gray-wolf algorithm applicable to the IIoT adaptive trust service coordination problem driven by QoS, two mechanisms are introduced in this paper that are based on the basic gray-wolf algorithm. The two mechanisms are as follows:

(1) Pareto archive. The gray-wolf algorithm generates new solutions in every iteration, so it is necessary to use the Pareto archive to store the Pareto solution in these new solutions. When the number of Pareto solutions exceeds Nbp, which indicates the size of the archive, the archive will be clipped according to the crowding distance.

(2) Storage space renewal of the Pareto solution set. (a) When a new solution can control one or more solutions in the file, the dominated solution will be eliminated so that the new solution can enter the archive. (b) If the new solution and archive members cannot dominate with each other, the new solution should be added to the archive. (c) If the archive is full, the similarity between the dominating schemes will be calculated using the Euclidean distance, finding two or more schemes with higher similarity and omitting one of them. The similarity of the new solution should be low to improve the diversity of the final approximate Pareto-optimal front.

The trust service coordination of the IIoT is quantified as the location where the individual gray wolf approaches the prey in the multi-objective gray-wolf algorithm, in which one location corresponds to one service coordination scheme. The role of the leader wolf is to control the direction of motion of the gray wolves and the real-time location, thereby improving the service coordination, meeting the requirements of service compositions, and finally providing the optimal solution. Specifically, Algorithm 2 is shown as follows.

**Algorithm 2.** Multi-Objective Gray-Wolf Algorithm for IIoT service coordination**Input:** The QWS [28] data set was collected by Al-Masri and Mahmoud of the University of Guelph, which contains 2507 actual service attribute parameters such as the response time, availability, throughput, and so on. However, this data set lacks the data of trust. To expand the QWS data set, trust data sets were generated via a simulation experiment based on the trust evaluation model in this paper. **Output:** A set of Pareto sets related to the service composition of industrial networking, and the solution set is judged according to the four indexes in the final. **Begin:** Calculates the wolf’s real-time location (corresponding to the service index of the service composition).1 Initialize the gray-wolf population $X_{i}$ (*i* = 1, 2, ..., *n*)2 Initialize a, A and C3 Calculate the fitness of each search agent4 Find non-dominated solutions and initialize the archive with them5 Calculate $X_{\alpha },\;X_{\beta },\;X_{\delta }$
6 Add alpha and beta to the archive7 T = 1;8 while (*t* < Max number of iterations)9  for each search agent10   Update the position of the present search agent by equation11  end for12  Update a, A, and C13  Calculate the objective value of all search agents14  Find solutions that are not dominated15  Update archive16  if archive is full17   Run the similarity mechanism to omit one of the present archive members18   Add the new solution to archive19  end if20  if any newly added solution in the archive is beyond the hypercube21   Update grid to cover new solutions22  end if23 Update $X_{\alpha },\;X_{\beta }$, $X_{\delta }$
24 Add alpha and beta to archive25  T = t + 1;26 end while27 return archive

## 4. Experiments and Discussion

Based on the MATLAB tool, this paper verified the convergence and accuracy of the proposed adaptive malicious rating filter algorithm based on rating sets and trust evaluation mechanism. Then, the MOGWO algorithm was used to solve the adaptive trust service coordination problem in the QoS-driven IIoT and was compared with the commonly used Dragonfly [29] and particle swarm optimization (PSO) [30] algorithms to verify its advantages for solving the large and complex search space in the IIoT trust service coordination.

### 4.1. Experiments of the Adaptive Trust Evaluation Model

#### 4.1.1. Experimental Environment

In the experiment, a node represents a service and the state of each node is dynamic. The meanings of the input parameters are shown in Table 1.

The number of heterogeneous intelligent objects/devices that provide different service in IIoT environments is $N_{P}=200$ and the proportion of malicious nodes is λ=30%, which is relatively high in IIoT enviroments. The suspicion threshold of the malicious ratio and service times for judging malicious raters are $\hat{MP}=0.7$ and $C_{i}=6$, respectively. The initial trust value of all devices is set to 0, which indicates ignorance. The purpose of the experiment is to show that when the malicious raters in the service composition perform the various attacks mentioned above, the proposed algorithm can quickly judge malicious raters and blacklist them. At the same time, benign raters will not be misjudged.

#### 4.1.2. Experimental Results and Analysis

(1) First, we study the performance of the adaptive malicious rater filter algorithm based on rating sets under bad mouthing and ballot stuffing attacks. When H(x) takes different functions, the speed of convergence, accuracy, and judging error during the recognition process are compared. The function H(x) is the threshold function for judging malicious raters, whereas the substitution for x is the variance $\sigma_{i}$ of the rating set.

In this experiment, the ratio of malicious nodes λ is 30% and the interaction period T is 25 interactions. We define *H*(*x*) using three functions: x, $\sqrt{x}$, and $\sqrt[{3}]{x}$. Next, we determine the coefficients for the functions. Through experiments, we found that the judging error is the smallest when the coefficients are 2.5, 1.5, and 1.3, respectively, and the convergence speed is also the fastest. Therefore, in the following experiments, we compare the performance of the following functions: 2.5x, $1.5\sqrt{x}$, and $1.3\sqrt[{3}]{x}$.

**Figure 4:**
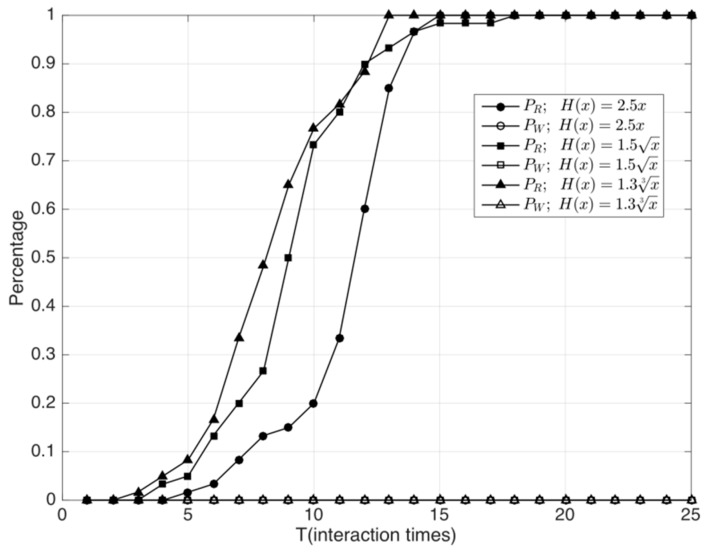
Performance evaluation and comparison of different H(x) functions.

From Figure 4, we observe that by using this algorithm, the misjudging rate of the three functions is always 0, as can be seen on the *x* axis, which indicates that when λ = 0.3, the judging error of this algorithm is zero. Because of the suspicion threshold of service times in the algorithm, the recognition rate of malicious nodes is zero in the first few interaction rounds. When the recognition rate of the algorithm increases gradually as the number of interaction rounds increases, we find that $1.3\sqrt[{3}]{x}$ is the fastest function and 2.5x is the slowest in terms of convergence speed of recognition rate.

(2) In this section, we study the performance of our trust evaluation system under the opportunistic service attacks. In experiments, the proportion of malicious nodes λ is also 30%. To achieve a slower rate of increase and a faster rate of decrease in the trust value, we set α and β as 0.25 and 2, respectively.

**Figure 5:**
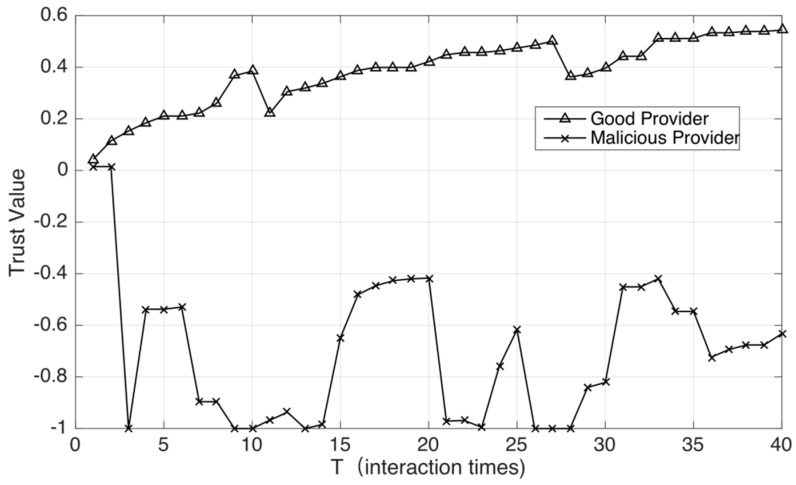
Trust evaluation of opportunistic service providers and benign service providers.

In Figure 5, we select a benign service provider and an opportunistic service provider for comparison. By comparison, we find that because the opportunistic service providers intermittently provide benign services and malicious services in the system, their trust value shows violent fluctuation. However, their trust value is always much lower than that of benign service providers. Therefore, our trust evaluation mechanism has also proved to be effective under this type of attack mode.

(3) We consider the situation in which malicious attackers in the IIoT perform hybrid attacks, which means that they perform bad mouthing attacks, ballot stuffing attacks, and opportunistic service attacks simultaneously. In the experiment, we still set λ to 0.3 and compare the performances of $1.5\sqrt{x}$ and $1.3\sqrt[{3}]{x}$.

**Figure 6:**
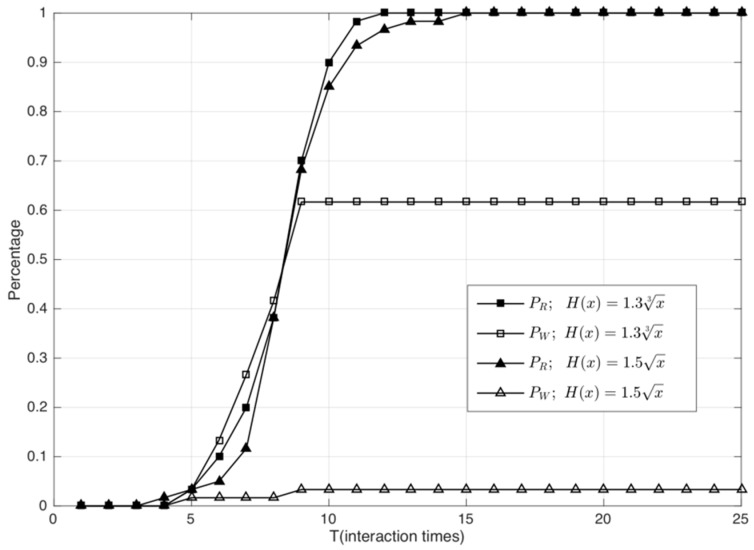
Performance comparison of $1.5\sqrt{x}$ and $1.3\sqrt[{3}]{x}$ in hybrid attacks.

In Figure 6, we observe that when using 1.3$\sqrt[{3}]{x}$, the rate of judging error exceeds 60%, which indicates that 1.3$\sqrt[{3}]{x}$ is not applicable in hybrid attacks. Although the convergence speed of $1.5\sqrt{x}$ is slightly slower than that of $1.3\sqrt[{3}]{x}$, its judging error is always less than 5%. By comparison with 2.5x and $1.3\sqrt[{3}]{x}$, we can conclude that $H\left(x\right)=1.5\sqrt{x}$ is the most robust function in our proposed model. Then, we observe the trust evaluation performance of the system under the hybrid attacks when $H\left(x\right)=1.5\sqrt{x}$.

**Figure 7:**
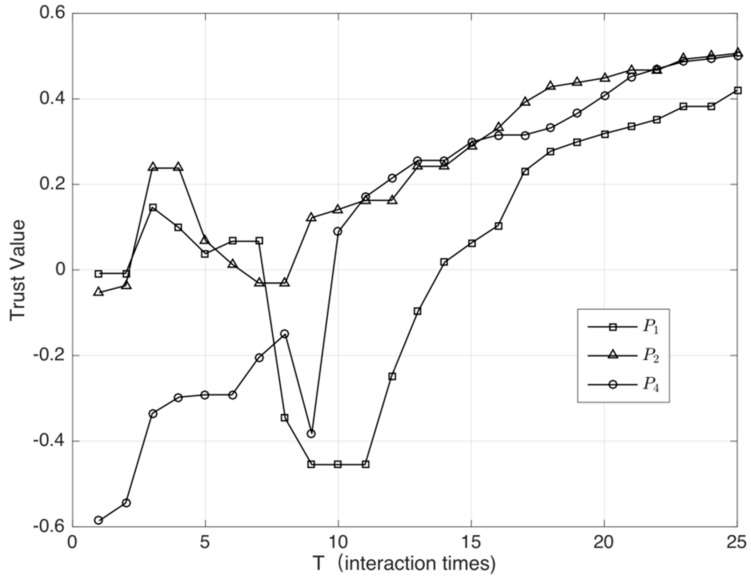
Trust value curve of the benign node under hybrid attacks.

In Figure 7, we select three benign nodes ($P_{3}$ is a malicious service provider in this experiment) and plot their trust value curve. In T∈[5,10], because of attacks from malicious service providers, the trust evaluation of benign nodes is greatly affected. However, after the proposed algorithm accurately filters the malicious nodes, the benign node’s trust value can still converge accurately and quickly.

The anonymity and tamper-resistance of blockchain play an important role in the overall process of trust value maintenance. To begin with, the anonymity of blockchain guarantees the authenticity and objectivity of P2P ratings. Moreover, the validity of trust evaluation relies on the tamper-resistance of blockchain, because the trust value derived by our trust evaluation mechanism is personal and individual, instead of P2P in the SIoT.

### 4.2. Experiments of Trust Service Coordination Model

#### 4.2.1. Experimental Environment

To verify the effectiveness of the gray-wolf algorithm for solving the adaptive trust service coordination in the industrial IoT, the parameters of gray-wolf algorithm are set as follows: the population size is 100; the maximum number of iterations is 100; the number of running algorithms is 100; the number of optimal solutions of the service coordination is 100; the candidate service dataset is four-dimensional (response time, availability, throughput, reliability) and each subtask has the same number of candidate services. The performance evaluation of service composition optimization is based on four indicators. When the above constraints are satisfied, perform the simulation experiments and compare the results of the algorithm with those of the dragonfly algorithm and the particle swarm optimization algorithm. We used the QWS datasets and the trust dataset derived from the proposed trust mechanism in the experiment. For the datasets satisfying the constraints, the usability, response time, accessibility, and throughput are normalized and the trust service coordination problem is transformed into the maximum fitness problem.

#### 4.2.2. Experimental Results and Analysis

In the multi-objective optimization algorithm, a set of optimum solutions can be obtained by applying the Pareto optimality theory. To verify the effectiveness of the improved MOGWO for service coordination in the industrial IoT, we evaluate its performance by comparing the means and the extreme values of Pareto solutions with those of the multi-objective particle swarm optimization (MOPSO) and the multi-objective dragonfly algorithm (MODA).

**Figure 8:**
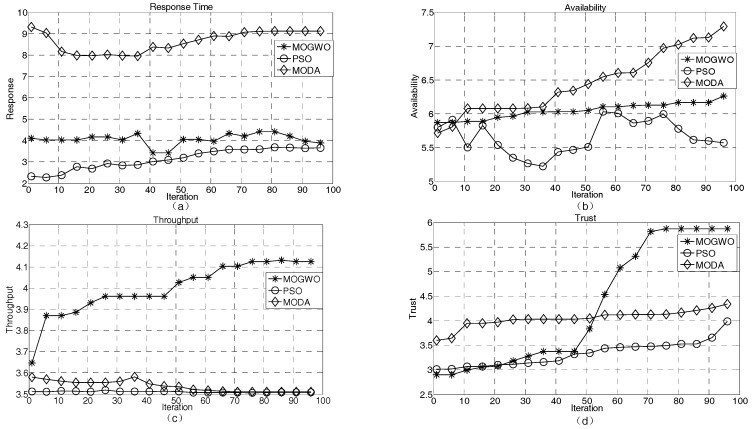
Algorithm mean comparison. (**a**) response time; (**b**) availability; (**c**) throughout; (**d**) trust. MOGWA—multi-objective gray-wolf optimizer; PSO—particle swarm optimization; MODA—multi-objective dragonfly algorithm.

Figure 8 shows that the mean values of the QoS indexes of the Pareto solution set of MOGWO, MODA, and PSO algorithms vary with increasing iteration number under the premise of 10 subtasks of the service coordination. The abscissa in Figure 8 represents the number of iterations and the ordinate represents the value of the corresponding indicator. As seen from Figure 8a, in the early iterations, the response time of PSO is the best and that of MODA is the worst; in the iterative process, the trend of the improved MOGWO and PSO is relatively stable, whereas the slope of the MODA curve is larger; in the late iterations, the three algorithms gradually converge. Therefore, the application of the MOGWO and PSO algorithms in service coordination meets the users’ demands for the response time. As shown in Figure 8b, MODA tends to be more available as the number of iterations increases, which is more consistent with the requirements of service coordination for availability metrics. The availability metrics for the MOGWO and PSO solutions are comparatively poor, but more stable. From Figure 8c, we see that MOGWO’s Pareto solution set obviously has more throughput and can provide more users at the same time in unit time. From Figure 8d, we see that the MOGWO algorithm has a poor reliability in the early iterations, but as the number of iterations increases, it gradually outperforms the PSO and MODA algorithms in terms of the reliability index and converges in the later iterations. Overall, the improved MOGWO algorithm is relatively stable during iterations. In terms of response time, throughput, and reliability indicators, MOGWO outperforms PSO and MODA, and, therefore, is more suitable for meeting the requirements of IIoT QoS coordination.

**Figure 9:**
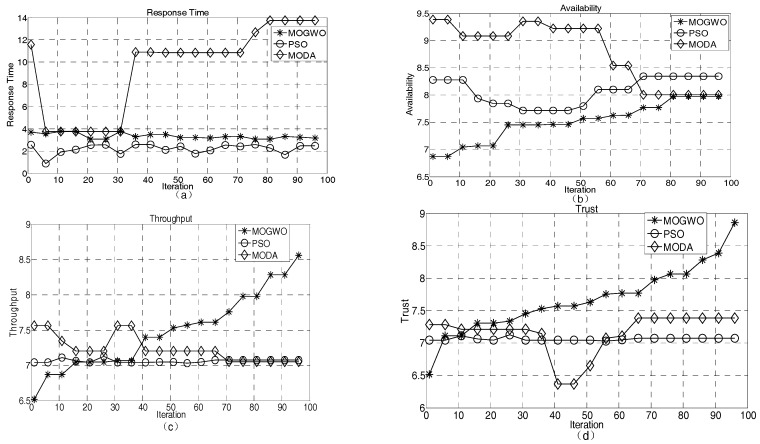
Comparison of the optimal value of a single index. (**a**) response time; (**b**) availability; (**c**) throughput; (**d**) trust.

Figure 9 shows the variation of a single indicator as the number of iterations increases. In Figure 9, the horizontal axis represents the number of iterations and the vertical axis represents the corresponding index. During service coordination, users often choose the best performing set of services. However, because of different user preferences for indicators in different applications, to better meet user requirements for service coordination QoS, four evaluation indexes need to be compared one by one. From Figure 9a, we see that the minimum response time of the MOGWO Pareto solution is relatively stable and is obviously better than that of the MODA algorithm, but worse than that of the PSO algorithm. From Figure 9b, we know that the MOGWO usability index is slightly worse than those of the MODA and PSO algorithms. From Figure 9c, the throughput index of MOGWO is poor in the early iterations, but is obviously better than those of the PSO and MODA algorithms in the later iterations. The goal of service coordination is to serve more users at the same time. As seen from Figure 9d, with an increasing number of iterations, the reliability index of MOGWO gradually becomes better than those of the PSO and MODA algorithms. In terms of overall performance, the improved MOGWO algorithm is more consistent with the QoS requirements of the IIoT QoS coordination.

## 5. Conclusions

In this paper, we consider the issue of QoS demand-driven IIoT services mismatching. In order to improve the quality of Pareto-efficient service resource allocation, we need to improve the trust of the service to meet the personalized QoS requirements of users. We propose a methodology of QoS-driven adaptive trust service coordination using MOGWO, which can quickly filter malicious service providers and improve the quality of service coordination well in the industrial internet of things, increasing not only the credibility but also the success rate. To meet different users’ requirements in QoS-Pareto service resource allocation, we can incorporate some evaluation indexes, such as response time, availability, and throughput. From our observations, a limitation of our approach is that the model considers only the vertical service dimension relationships (such as time, reliability, etc.) of the manufacturing service chain at the upstream, middle, and downstream. However, it does not consider the horizontal collaborative relationship between the service and product and enterprise dimensions. One direction for our future work would, therefore, be to build a new aspect-oriented social collaborative multi-dimensional QoS adaptation model, considering the service dimension not only of vertical but also of horizontal, in order to enable better accuracy of the adaptation model. Another limitation of our approach is that the MOGWO algorithm convergence is poor. We thus plan to develop an algorithm that considers the MOGWO algorithm combined with other algorithms to optimize and improve the convergence of the algorithm.

## Figures and Tables

**Table 1:** The parameters used in the paper.

Symbol	Meaning	Type
$N_{P}$	number of IIoT service providers	input
*T*	average interaction inter-interval time	input
λ	percentage of malicious service providers	input
α	parameter of trust increasing	design
β	parameter of trust decreasing	design
$\hat{MP}$	threshold of suspicion percentage	design
$C_{i}$	threshold of service times	design
H(x)	threshold function in the proposed algorithm	design
$T_{i}\left(t\right)$	trust value of $P_{i}$ at time t	derived
$P_{R}\left(t\right)$	recognition rate at time t	derived
$P_{W}\left(t\right)$	misjudging rate at time t	derived
